# When Disaster Strikes, Area Agencies on Aging Help Older Adults Prepare and Respond

**DOI:** 10.1093/geroni/igaf122.4324

**Published:** 2025-12-31

**Authors:** Traci Wilson

**Affiliations:** USAging, Washington, District of Columbia, United States

## Abstract

Global climate change has increased the frequency and intensity of natural disasters, which can have a disproportionate impact on older adults. According to data from a new module in the 2025 National Survey of Area Agencies on Aging (72% response rate) 65% of AAAs reported that a community within their service area has experienced a significant emergency or disaster within the past five years. This presentation will share nationally-representative data about the ways in which AAAs support older adults to prepare for emergencies, help the community prepare to respond to consumers, and ensure consumer safety. AAAs support individual emergency preparedness through providing educational resources (67%), addressing emergency preparedness as a standard part of the care planning process (45%), and maintaining a prioritized consumer registry (44%). AAAs also helps the community prepare to respond to consumer needs during disasters: 63% are involved in emergency planning collaboratives, 47% require providers to have emergency response plans in place, 45% are in regular two-way communication with EMS. The data will be supported by case examples describing how AAA emergency preparedness and response plays out before, during and after a natural disaster, including lessons learned.

